# Supplementary material for: Qualitative study of the roles of midwives in the provision of sexual and reproductive healthcare services in the Somaliland health system
Source: BMJ Open. 2023 Mar 14;13(3):e067315. doi: 10.1136/bmjopen-2022-067315 (PMC10030797; doi:10.1136/bmjopen-2022-067315)
Supplement: Supplementary data [file bmjopen-2022-067315supp001.pdf]

Appendix 1

Focus Group Discussion guide

1. What are your views and perceptions as a midwife in terms of your role in family planning (FP) and Post Abortion Care (PAC)?

2. What are the responsibilities and roles of midwives in relation to FP and PAC?

3. What are the challenges you face in providing these services (FP and PAC)?
    - What are the legal and socio-cultural frameworks for providing these services in your community?
    - In what ways do these frameworks impact you in your role as a midwife?

4. What support or resources do you feel would be most helpful for you to better fulfill your roles?

5. Is there anything else you would like to add or discuss related to your experiences as a midwife in Somaliland?
