# Supplementary material for: Qualitative study of the roles of midwives in the provision of sexual and reproductive healthcare services in the Somaliland health system
Source: BMJ Open. 2023 Mar 14;13(3):e067315. doi: 10.1136/bmjopen-2022-067315 (PMC10030797; doi:10.1136/bmjopen-2022-067315)
Supplement: Author's manuscript [file bmjopen-2022-067315.draft_revisions.pdf]

BMJ Open

BMJ Open is committed to open peer review. As part of this commitment we make the peer review history of every article we publish publicly available.

When an article is published we post the peer reviewers' comments and the authors' responses online. We also post the versions of the paper that were used during peer review. These are the versions that the peer review comments apply to.

The versions of the paper that follow are the versions that were submitted during the peer review process. They are not the versions of record or the final published versions. They should not be cited or distributed as the published version of this manuscript.

BMJ Open is an open access journal and the full, final, typeset and author-corrected version of record of the manuscript is available on our site with no access controls, subscription charges or pay-per-view fees (http://bmjopen.bmj.com).

If you have any questions on BMJ Open's open peer review process please email info.bmjopen@bmj.com

BMJ Open

A qualitative study of the roles of midwives in the provision of sexual and reproductive health services in the Somaliland health system.

Journal:	*BMJ Open*
Manuscript ID	bmjopen-2022-067315
Article Type:	Original research
Date Submitted by the Author:	09-Aug-2022

Complete List of Authors:	Maregn, Rahel Tesfa; Dalarna University, School of Health and Welfare<br>Bourret, Kirsty; Karolinska Institute, Department of Women and Children’s Health; McMaster University, Department of Obstetrics and Gynecology<br>Egal, Jama Ali; Dalarna University, School of Health and Welfare; University of Hargeisa College of Medicine and Health Sciences, Faculty of Nursing and Midwifery<br>Esse, Amina; University of Hargeisa College of Medicine and Health Sciences, Faculty of Nursing and Midwifery<br>Mattison, Cristina; McMaster University, Department of Obstetrics and Gynecology; Karolinska Institute, Department of Women and Children’s Health<br>klingberg-allvin, marie; Dalarna University, School of Health and Welfare; Karolinska Institute, Department of Women and Children’s Health

Keywords:	QUALITATIVE RESEARCH, REPRODUCTIVE MEDICINE, SEXUAL MEDICINE, PUBLIC HEALTH, Health & safety < HEALTH SERVICES ADMINISTRATION & MANAGEMENT

SCHOLARONE™ Manuscripts

I, the Submitting Author has the right to grant and does grant on behalf of all authors of the Work (as defined in the below author licence), an exclusive licence and/or a non-exclusive licence for contributions from authors who are: i) UK Crown employees; ii) where BMJ has agreed a CC-BY licence shall apply, and/or iii) in accordance with the terms applicable for US Federal Government officers or employees acting as part of their official duties; on a worldwide, perpetual, irrevocable, royalty-free basis to BMJ Publishing Group Ltd ("BMJ") its licensees and where the relevant Journal is co-owned by BMJ to the co-owners of the Journal, to publish the Work in this journal and any other BMJ products and to exploit all rights, as set out in our licence.

The Submitting Author accepts and understands that any supply made under these terms is made by BMJ to the Submitting Author unless you are acting as an employee on behalf of your employer or a postgraduate student of an affiliated institution which is paying any applicable article publishing charge ("APC") for Open Access articles. Where the Submitting Author wishes to make the Work available on an Open Access basis (and intends to pay the relevant APC), the terms of reuse of such Open Access shall be governed by a Creative Commons licence – details of these licences and which Creative Commons licence will apply to this Work are set out in our licence referred to above.

Other than as permitted in any relevant BMJ Author's Self Archiving Policies, I confirm this Work has not been accepted for publication elsewhere, is not being considered for publication elsewhere and does not duplicate material already published. I confirm all authors consent to publication of this Work and authorise the granting of this licence.

For peer review only

A qualitative study of the roles of midwives in the provision of sexual and reproductive health services in the Somaliland health system

Rahel Tesfa Maregn[1*] Kirsty M. Bourret[2,3], Jama Ali Egal[1,4], Amina Esse[4], Cristina A. Mattison PhD[2,3], Marie Klingberg-Allvin[1, 2*]

[1] School of Health and Welfare,
Dalarna University, Falun, Sweden

[2] Department of Women and Children's Health,
Karolinska Institute, Stockholm, Sweden

[3] McMaster Midwifery Research Centre
Department of Obstetrics and Gynaecology
McMaster University, Toronto, Canada

[4] Faculty of Nursing and Midwifery
College of Medicine and Health Sciences
University of Hargeisa, Hargeisa, Somaliland

*Corresponding authors:
Rahel Tesfa Maregn
School of Health and Welfare,
Dalarna University
791 88, Falun, Sweden
e-mail: h20rahem@du.se

Marie Klingberg-Allvin
e-mail: marie.klingberg-allvin@ki.se
Cell phone: +46 707174414

Wordcount: 4000

For peer review only - http://bmjopen.bmj.com/site/about/guidelines.xhtml

ABSTRACT

Objectives: To explores midwives´ perspectives in the provision of sexual reproductive health and right services in the Somaliland health system.
Methods: An exploratory qualitative design using focus group discussions (n = 6) was used. The study was conducted in the capital of Somaliland, Hargeisa, at six maternal and child health care centres that provide SRHR services. Qualified midwives (n=44) who had been working in the MCHs for a minimum of one year were recruited to participate, and only one did not participate due to illness
Results: The results highlight the intersection of gender with professional, socio-cultural, and legal factors that impact the role of midwives in the Somaliland health system. This was visible in five key areas: 1) post-abortion care, 2) family planning, 3) sexual and gender-based violence, 4) female genital mutilation/cutting, and 5) assisting women with unintended pregnancies. Midwives face multiple challenges due to resource constraints, a lack of clear, written guidelines that define their professional role, and limited occupational support for their mental health.
Conclusion: Our findings show how midwives play a crucial role in meeting and safeguarding the SRHR service needs of women and girls. They also address how midwives create solutions to address critical gaps within culturally restrictive and low-resource settings. Furthermore, we provide concrete ways in which their role crosscuts legal, cultural, and social spheres. Future research is needed to examine how midwifery expertise can be acknowledged and integrated into health policy to support evidence-informed paths for midwifery.

Key terms: sexual reproductive health, midwives, qualitative study, health systems research, Somaliland

For peer review only

For peer review only - http://bmjopen.bmj.com/site/about/guidelines.xhtml

STRENGTH AND LIMITATIONS OF THIS STUDY

- The main strength of our study is the richness of the data, which was collected by two professional Somali women with midwifery backgrounds, coupled with a collaborative approach throughout the research process between the research team in and outside of Somaliland.
- While there was much sharing during the focus group discussions, the setting may have limited how much individuals shared, as some may not have wanted to disclose personal details of their experiences in a group setting, especially given the sensitive topic areas.

For peer review only

For peer review only - http://bmjopen.bmj.com/site/about/guidelines.xhtml

Introduction

Sexual and reproductive health and rights (SRHR) interventions benefit women, girls, children, and societies at large and are considered a cornerstone to achieving universal health coverage (UHC) and reaching the Sustainable Development Goals (SDGs).[1,2] Following a life-course approach, the minimum standards of an essential package of SRHR services include contraceptive counselling; induced abortion, post-abortion care (PAC), contraception, antenatal, intrapartum, and postnatal care; and detection and prevention of sexual and gender-based violence (SGBV).[2,3] The provision of these essential SRHR services would contribute to reducing the global maternal mortality ratio to fewer than 70 per 100,000 live births and attaining the target of universal access to SRHR, as set out in the 2030 Agenda for Sustainable Development.[4] Midwives who are educated and regulated according to international standards are an essential part of the health system and can meet 87% of the population's SRHR needs.[5] Midwife-led care, supported by an enabling environment, could save an estimated 4.3 million lives annually by 2035.[6] While scientific evidence shows that midwives are vital to the delivery of safe and effective SRHR services, there are significant challenges in the provision of these services within and across countries due to social and cultural factors, gender inequalities and a lack of understanding of the role of midwifery in health systems.[3,7-9]

In 1991, Somaliland became a sovereign and independent region of Somalia. In a global comparison, this region suffers high maternal mortality rates, at an estimated 396 deaths per 100,000 live births every year.[10] The leading causes of maternal deaths are postpartum haemorrhage, pre-eclampsia/eclampsia, prolonged and obstructed labour, and infections combined with limited access to SRHR services.[10-12] The recent Somaliland Health and Demographic Report (2020) showed that 67% of children are delivered at home, further contributing to high maternal and neonatal mortality and morbidity. Moreover, cultural practices such as female genital mutilation/cutting (FGM/C) are prevalent. Approximately 98% of women and girls between the ages of 15–49 have undergone the procedure, 61% of whom underwent Pharaonic circumcision, a severe form of FGM/C. Access to contraceptives is limited, with 7% of married women reporting practising birth spacing, and only 1% of these are using modern contraceptives.[10]

The limited availability of healthcare facilities and the extreme shortage of qualified healthcare professionals further impact health outcomes.[10,11,13] Despite these shortfalls, midwifery institutions and universities in Somaliland follow the International Confederation of Midwives' (ICM) standards for midwifery education to meet global requirements and improve the quality of SRHR.[13,14] There are 1313 midwives in Somaliland (993 with diploma certificates, 270 with Bachelor's degrees, and 50 with Master's degrees in midwifery) (Somaliland Nursing and Midwifery Association, 2021). Due to the shortage of healthcare professionals and midwives, low-resource settings like Somaliland often struggle with providing pregnant women with complete health coverage. As a result, women living in these settings rely on non-professional traditional birth attendants who have acquired skills by assisting mothers during home-based childbirths

For peer review only - http://bmjopen.bmj.com/site/about/guidelines.xhtml

without any medical training.[10-12,15] Our study aimed to explore the role of Somaliland midwives in the provision of SRHR services within the health system.

Methods

We followed an exploratory qualitative study design and used focus group discussions (FGDs) with midwives for data collection.[16,17] The data was analysed inductively using thematic analysis.[18] Since this study is the first of its kind in Somaliland; this design allowed us to understand and describe the nature of the problem.

Data Collection

The study was conducted in the capital of Somaliland, Hargeisa, at the Digaale, Jimcaale, Sh Nurr, Sh Umar, Saxardid, and Iftin Maternal and Child Health Centres (MCHs). These MCHs (n = 6) are spread out across Hargeisa and provide services to internally displaced people and host communities. All the MCHs in Somaliland provide birth spacing, PAC, antenatal, intrapartum, and postnatal care by trained midwives. The MCHs are monitored and evaluated by the Somaliland Ministry of Health and Development.

A purposive sampling approach was used to recruit practising midwives from the six MCHs. Qualified midwives who met the inclusion criteria had been working in the MCHs for a minimum of one year and had experience with SRHR service provision. A total of 44 midwives were recruited to participate via face-to-face invitations, and only one did not show up due to illness. The participants were given oral and written information about the study before consent. Six FGDs were conducted in the respective MCHs between January and February 2021. The research team designed an FGD topic guide to facilitate discussion and aid in data collection.[16,17] The guide was piloted in one of the MCHs with seven participants, after which a few probing questions were added. All the FGDs were facilitated by JAE, assisted by AE, and conducted in-person in the Somali language, with the exception of one that was conducted in English. Each FGD took approximately one and a half hours.

Analysis

The audio recordings were transcribed and translated into English by a Somali translator. The transcribed data was checked for accuracy (JAE) and de-identified. The data were then inserted into NVivo-12, where it was inductively arranged and coded.[18] A thematic analysis approach was applied (ibid), which resulted in a codebook of emerging themes and sub-themes. Feedback was obtained from the participants after sharing the results.

Patient and public involvement

None.

Results

In the analysis, two main themes were identified: 1) the Somaliland context and its impact on midwives' role in SRHR (i.e., midwives working environment at the intersection of health, socio-

For peer review only - http://bmjopen.bmj.com/site/about/guidelines.xhtml

cultural and legal systems); and 2) midwives' perceptions of their role in broader SRHR in the context of Somaliland. Each of these included related sub-themes (Table 1). Context-specific terminologies were used, such as child spacing/birth spacing (defined as family planning or contraceptive use) and illegal pregnancy (means the pregnancy has been conceived outside of marriage). The following sub-themes elaborate on how the midwives described the lack of occupational and legal support in their work environments and the tensions they experienced between socio-cultural norms and their professional role.

Table 1: Condensed meaning units and selected quotes from the results showing the data extraction process.

Themes	Subthemes	Generic codes	Selected quotes
**Somaliland context and its impact on midwives' role in SRHR services**	*Midwives' navigation of health system barriers*	Service improvisation with available resources	If a woman is bleeding severely, we ensure there is canula access, and we transfer them to the hospital using the team leader's personal car, as the MCH has no ambulance, which we really need. (Focus Group 5)
		Need for in-service training	We, midwives, need more training and experience. We would have a lot of impacts. (Focus Group 6)
		Service provision in unsafe working environments	Some husbands would, without a doubt, come to us carrying a gun if they ever found out we gave their wives contraceptives without their consent. (Focus Group 3)
		Need for occupational support	We, as midwives, need psychological support to deal with our own trauma; only then can we support women. (Focus Group 3)<br>Midwives don't make enough money to sustain their lives, so a lot of them are working double jobs and shifts. There is no monitoring or evaluation from the Ministry as well. (Focus Group 6)
	Professional dilemmas within civil, cultural, and legal frameworks	Husbands' consent for family planning and post-abortion care	There is no law that says anything about family planning. It is just that, as this is a family matter, it should be encouraged for the husband and wife to discuss these things and for no one to interfere. People always say that no husband would refuse something that is good for his wife who he loves and cares

For peer review only

For peer review only - http://bmjopen.bmj.com/site/about/guidelines.xhtml

			about, but we know that it is not that simple. (Focus Group 6)
		Unsafe abortion practices	Here in Somaliland, there are a lot of unsafe abortions, which causes high mortality rates. (Focus Group 6)<br>A lot of people do it (abortion), but it's not legal. Some of them carry the baby for nine months and then throw away the baby. There is no legal abortion at all in Somaliland. (Focus Group 5)
		Families dealing with sexual violence internally	There are a lot of people who get away with those kinds of crimes by giving out money or because of who they are based on their clan or their wealth. (Focus Group 6)
		Provision of family planning	Family planning is part of our services, and we tell every mother that comes to the health facility about child spacing. (Focus Group 2)
	Provision of family planning and post-abortion care	Provision of post-abortion care	We do post-abortion care, we got training. We have misoprostol. We use misoprostol. (Focus Group 1)<br>Most of them need further care, including a blood transfusion. But if she has had a complete abortion that can be identified by ultrasound, we give her counseling about family planning. (Focus Group 2)
**Midwives' perceptions of their role in broader SRHR**		Sociocultural norms and FGM/C	Most mothers believe that their daughters will not be accepted for marriage if they don't have a Pharaonic circumcision. This also includes most men. They say the girl is not pure. (Focus Group 5)
	FGM/C and sexual and gender-based violence	Educating about FGM/C and treating post-FGM/C complications	When we have mothers come to us about other issues, like breastfeeding, we use these opportunities to educate them about the issues and problems FGM can cause. (Focus Group 3)
		Providing care for survivors of sexual and	Every week we come across two to three cases of rape, and most of those are kids being raped by family members. (Focus Group 6)

For peer review only

	gender-based violence	All women have the same options, regardless of where they come from. (Focus Group 6)
Advocate and system navigator for clients	Advocate and system navigator for clients	We all used it and advised each other about it (family planning). (Focus Group 4)<br>We are lucky that we have religious evidence that encourages family spacing; otherwise, things would be worse today. We always use this to our advantage to support mothers. (Focus Group 5)

The Somaliland context and its impact on midwives' role in SRHR

Midwives' navigation of health system barriers

The midwives stated that current health system arrangements limited their scope of practice, threatened their safety, and subsequently impacted the provision of quality of care. Examples included a general lack of infrastructure; a lack of care guidelines, including definitions of tasks and midwives' professional role; inadequate service delivery arrangements; a lack of occupational support and safety; and psychological distress due to personal experiences with FGM/C.

> *There is no legal framework [concerning family planning services] in Somaliland. This lack means healthcare workers do not get the support they need to support women regardless of what the husband says.* (Focus Group 5)

Regarding infrastructure, the participants emphasized frequent disruptions in equipment, pharmacological supplies, and emergency transport. A lack of contraceptives was seen as particularly detrimental, leaving many women exposed to the risk of unintended pregnancy. Furthermore, a lack of supplies meant that the midwives could not manage emergencies, and a lack of transport meant that instead of treating women, they became logisticians, using their own cars or collecting funds for taxi fees to escort women to referral hospitals.

> *We don't have a scanner in the MCH, so we have no way of assessing the level of care a woman needs here […]. If a woman is bleeding severely, we ensure that there is access to a canula, and we transfer them to the hospital using the team leader's personal car, as the MCH has no ambulance, which we really need.* (Focus Group 5)

For peer review only - http://bmjopen.bmj.com/site/about/guidelines.xhtml

According to the participants, challenges in service delivery arrangements, such as unclear interprofessional roles and responsibilities, high workloads due to a shortage of midwives, and overcrowded hospitals severely limit the quality of care and contribute to adverse outcomes.

The lack of access to trainings and inadequate hands-on supervision were found to have impacted the midwives' confidence in practising broader SRHR competencies within their scope. For example, not all midwives have been trained in post-abortion care or feel equipped to implement life-saving skills when faced with complications from incomplete abortions.

> *We need more support and training. We received training not so long ago, but without practical practice with real women, we cannot perform more than this.* (Focus Group 5)

Conversely, practising according to the formal midwifery scope of practice could lead midwives into unsafe circumstances. For example, contrary to cultural laws, it is within the profession's regulatory framework to provide contraception to women without male consent; however, midwives reported being put in unsafe positions if they did so. Verbal, physical, psychological, and legal threats from clients' family members and a lack of institutional protection were common occurrences in their day-to-day practices.

> *In the morning, the husband came into the MCH with a big wooden stick and said, 'Bring out the women in the staff—she will die, or I will.' The police were called, and the husband told the police personnel that the midwife had stopped the childbearing of his wife. There is no law that can guarantee or protect healthcare staff from issues like these.* (Focus Group 1)

In terms of psychological distress, all midwives in Somaliland are women, and most have undergone FGM/C. Midwives continue to suffer trauma from their own lived experiences with FGM/C. They described experiencing recurring traumatization in their day-to-day work by being exposed to women and young girls with FGM/C. They also described a lack of support for their mental well-being and a diminished capacity to manage their trauma after providing services to affected women and girls, especially those suffering complications from the procedure. There was a consensus across the focus groups that therapeutic support was essential, not only for midwives' mental health, but to also enable better care provision to their clients.

> *Every midwife needs to talk and find therapy to deal with these wounds from the past. It haunts me until today.* (Focus Group 1)

Professional dilemmas within civil, cultural, and legal frameworks

In Somaliland, there are currently no laws either allowing or prohibiting women from consenting to their own care management, however, cultural law dictates that either a husband or a male family

For peer review only - http://bmjopen.bmj.com/site/about/guidelines.xhtml

member must consent to the care, even in urgent or life-threatening circumstances. Midwives described how the blending of boundaries between cultural norms, customary traditions, and the legal system informed their decision-making and the care pathways of their clients. The midwives felt they were professionally and ethically required to provide services to their clients, yet both a midwife and their client could face serious consequences if they did not seek male consent.

> *[…] but legally, there is no law against giving any method to a woman.* (Focus Group 3)

These discussions were the most prominent regarding the provision of family planning, emergency post-abortion care, and gender-based sexual violence.

> *When a woman comes in with abortion complications, you can't help her without her husband. You need the husband present because you need him to sign the consent form, so you don't face any problems afterwards because anything is possible.* (Focus Group 6)

On the one hand, midwives are responsible for safeguarding the well-being of women, and on the other hand, they are part of a society where rape cases are hidden, pregnancy outside marriage is stigmatized, and law enforcement against perpetrators of sexual assault is not sought. Midwives in all six focus groups discussed how the complex intersections of their work environments, personal experiences, cultural norms, and laws impacted their personal and professional well-being and their capacity to provide quality care to women and girls.

Midwives' perceptions of their role in broader SRHR

When midwives were asked about post-abortion care and contraception, discussions organically led to FGM/C, sexual and gender-based violence, and how they advocated for womens' SRHR. The three subthemes are discussed below.

<u>Provision of post-abortion care and family planning services</u>

Despite the challenges and threats the midwives experienced daily, they agreed that they remained as the primary care providers for women needing post-abortion care and family planning. The extent of family planning services provided by midwives includes premedical assessment, family planning counselling, couples' mediation, nutritional support, breastfeeding guidance, and liaising and referrals based on women's needs. The midwives provided family planning methods if they were available. While it was within their role to provide post-abortion care, many described a lack of training, supervision, and supplies that limited their capacity. Only one of the MCHs had midwives who were trained and willing to provide post-abortion care; however, these midwives stated that there were few opportunities to practice.

For peer review only - http://bmjopen.bmj.com/site/about/guidelines.xhtml

> *We give women all the family planning information; unfortunately, we do not provide family planning in this MCH. Those who need family planning, we connect/transfer to other MCHs*. (Focus Group 1)

<u>FGM/C and sexual and gender-based violence</u>

FGM/C is a common practice in Somaliland, and participants linked its high prevalence to socio-cultural beliefs of gender norms, such as the importance of maintaining women's purity, increasing women´s marriage acceptance, and preventing pregnancy outside of marriage. It is important to note that in Somaliland, pregnancy outside marriage is illegal. The midwives discussed how the practice of FGM/C impacted their roles as both a midwife and a woman.

The midwives stated that their role included providing emergency treatment post FGM/C for bleeding and/or infection, providing routine intrapartum care, supporting women who experienced FGM/C, and educating women about SRHR concerning FGM/C.

> *As a midwife, I tell my clients the best way to prevent illegal behaviour is to educate your children in a proper way. We met many girls who had been circumcised and still have illegal pregnancies.* (Focus Group 2)

Woven into their narratives were the ways in which the midwives were psychologically and emotionally impacted by their own experiences with FGM/C when caring for women who had undergone FGM/C, which is a common occurrence given the extent of the practice. The midwives highlighted the importance of receiving mental health support to better care for themselves and their clients.

> *The woman who cut me really traumatized me. Even years after being cut, I remember getting goosebumps all over my body whenever I saw her. I had 10 people holding me down while I was being cut. Now that I know better, I can say that what she did to me was completely destructive and damaging. Even today, it bothers me to see someone in pain during childbirth. I was basically someone who had surgery without anaesthesia. I was being cut up, and it is still very traumatic.* **(**Focus Group 3)

In addition to their own traumas, the midwives described how socio-cultural expectations regarding FGM/C lead them to face ethically difficult scenarios in their professional lives, which at times means acting outside of their scope of practice. The midwives viewed these instances as a harm-reduction approach to the complications and long-term effects of FGM/C. The midwives also claimed that there has been a shift in the demand for FGM/C services from community circumcisers to MCHs. At MCHs, midwives face societal pressure from the families of girls to

For peer review only - http://bmjopen.bmj.com/site/about/guidelines.xhtml

provide FGM/Cs. Often, families cite the risks of turning to unsafe community circumcisers to coerce and manipulate midwives into providing FGM/C.

> *A woman said, 'I need circumcisions.' And said, 'If you refuse, I will get someone else to come in and do it for me.' I refused, and she tried to give me money to do that.* (Focus Group 1)

The midwives recognized that refusing to provide FGM/C would inevitably result in them having to treat girls with complications after they were taken to community circumcisers. The risk to the girls combined with their personal trauma compelled them to act, even going as far as performing Sunni circumcisions (described by the focus groups as cutting off the tip of the clitoris) as a way of protecting the girls from a more severe form of FGM/C, and its related complications.

The midwives supported reducing the practice overall, believing in awareness for prevention and taking opportunities to educate mothers about the cultural misconceptions and consequences of FGM/C. They explained that their role as educators and awareness creators brought them satisfaction, especially with regard to their personal experience with FGM/C. On the other side, midwives expressed feeling that the community saw them as playing contradictory roles by performing FGM/C yet promoting its decline.

> *Most healthcare providers have carried out FGM, especially the Sunni type, and they know the consequences of FGM. The reason they perform Sunni type is to prevent girls from undergoing the Pharaonic type […] How is it possible to encourage the community to stop FGM and, on the other hand, we continue Sunni type?* (Focus Group 2)

In the discussions, some midwives questioned the cultural norms behind FGM/C and its practice in the MCHs.

> *Where does this idea of making a girl 'halaal' (pure) even come from? [...] Well, if midwives are given rules to follow, they all need to obey those rules. So, rules need to be created. Holding midwives accountable and having consequences for their actions will make them listen.* (Focus Group 3)

Pregnancies resulting from sexual and gender-based violence and pregnancy outside of marriage, which is illegal in Somaliland, are highly stigmatized and often hidden by women and girls or resolved within families. Midwives provide frontline care to these women. The participants said they provided equal services without prejudice and gave examples of counselling women and girls who got pregnant outside of marriage. In some cases, the midwives mentioned advocating for and supporting rape survivors to prosecute their perpetrators.

For peer review only - http://bmjopen.bmj.com/site/about/guidelines.xhtml

> *We asked for an investigation and requested laws and regulations. In this specific case, we ended up winning, getting everyone off our backs, and finally sentenced the guy to jail for 20 years***.** (Focus Group 6)

<u>Advocate and system navigator for clients</u>

The midwives' gender intersected with their professional role, and some participants mentioned seeing themselves as role models and using their status to support other women. For example, they role-modelled the benefits of SRHR services, encouraging women to follow suit in their respective communities. They also believed that their own uptake of services could impact social norms to be more accepting and open to change. In addition to supporting women and each other professionally, they claimed to use religious evidence and education and involve trusted community members by forming male and female support groups, which helped them reach out to the community and make positive changes in SRHR. For instance, when husbands refuse to consent to family planning services, a midwife may use religious doctrines as evidence to support their actions, involve older adults to mediate between a couple, or sometimes provide confidential services to a woman without male consent. The midwives also created programs to teach women about their reproductive rights and create awareness of women's health within the community to empower women and increase their access to and use of SRHR services. They described this as a significant way to inform the community and raise awareness.

> *After the training is finished, they (fathers and mothers' groups) go around villages and look for people with problems. They work with us now and are so valuable and powerful in the community.* (Focus Group 1)

Discussion

To our knowledge, this is the first study to explore the role of midwives in the provision of SRHR in Somaliland, a post conflict setting, and from the perspectives of midwives. Participants described their role expanding beyond antenatal and delivery care to primarily include PAC, contraceptive counselling, SGBV, FGM/C, and counselling concerning unintended pregnancies (i.e., pregnancies out of wedlock). They also described how they navigate legal, cultural and social spheres, finding creative ways to mitigate and circumvent obstacles for women and girls in dire circumstances. Somaliland midwives face multiple challenges from a lack of formal arrangements, primarily written guidelines and policies, that explicitly define their role as healthcare professionals leaving them vulnerable to arbitrary persecution and other social consequences. They felt a lack of clinical guidance when managing intrapartum care for women with FGM/C. Furthermore, their lived experiences as women and survivors of FGM/C caused them to experience trauma and suffering in their day-to-day life with little or no mental health support. Under extreme mental stress and lack of safety, midwives are in a constant dilemma. Their decision-making process intersects with their professional, community, religious, and individual selves.

For peer review only - http://bmjopen.bmj.com/site/about/guidelines.xhtml

Similar studies from different contexts have also shown how midwives improvise strategies to provide emergency SRHR care when there is a lack of resources.[19,20] In the absence of clear guidelines and policies, healthcare professionals tend to employ their individual interpretation of social norms and laws.[21] Our results showed no consensus regarding appropriate strategies to address FGM/C practices. Some midwives attempted to deter communities from this socio-cultural norm by citing the sexual and reproductive health risks it poses to women and girls. Others argued that FGM/C should be performed in legal facilities to reduce adverse outcomes of unsafe procedures conducted by non-medical individuals outside clinical settings. Some felt coerced and forced to agree to families' requests. Similar studies in Somaliland and elsewhere have also highlighted the controversies surrounding FGM/C, showing a lack of reconciliation between the two sides of the FGM/C practice spectrum.[22,23]

Our results provide further insight into how midwives' personal gendered experiences in the Somaliland community and having experienced FGM/C may come into play, causing distress and influencing clinical decision-making. This includes services such as navigating out of wedlock pregnancies, providing PAC and supporting women to mitigate or navigate experiences of sexual and gender-based violence. Midwifery-led interventions, such as contraceptive counselling, are associated with efficient use of resources and a positive impact on womens' reproductive health outcomes.[6] Recent scientific evidence from similar settings shows midwives could play an important role in PAC treating first and second-trimester incomplete abortion using misoprostol.[24,25] Reports from Somaliland have highlighted that to increase women's access to evidence-based SRHR; midwives must be supported within the health system to broaden their scope of practice in correspondence to ICM Global Standards. The 2020 Somaliland Nursing and Midwifery Association's report posits that midwives contribute to improving overall population health and empowering individuals and communities.[26] Midwives in our study clearly state that to continue their work, they require the resources to manage the complexity of the experiences women and girls in Somaliland face regularly. They asked that they urgently receive trauma-informed mental health care so they can provide trauma-informed care to women, girls, and their communities. While our results highlight the positive impact midwifery-led care for women and girls, they demonstrate the urgent need to invest in structural supports for the profession.

The main strength of our study is the richness of the data, which was collected by two professional Somali women with midwifery backgrounds, coupled with a collaborative approach throughout the research process between the research team in and outside of Somaliland. Research generated by midwives is needed to create evidence-informed policies to support the integration of the profession into health systems.[9,27,28] While there was much sharing during the FDGs, the setting may have limited how much individuals shared, as some may not have wanted to disclose personal details of their experiences in a group setting, especially given the sensitive topic areas.

Conclusion

For peer review only - http://bmjopen.bmj.com/site/about/guidelines.xhtml

Maternal mortality and morbidity remain a significant challenge in Somaliland, especially in relation to services that are highly stigmatized, such as contraception, abortion care (including post-abortion care) and sexual and gender-based violence. Midwives are trusted members of the community and are often the first point of care for such services. They truly are integral to providing women and girls with safe, equitable SRHR. Our research provides a rich portrayal of midwives who are essential to the provision of equitable SRHR, yet are not fully supported by policies, laws, or institutions, often living in fear of the consequences of their behaviours. The United Nations Population Fund has highlighted four key investment areas to improve the integration of midwives in health systems 1) health workforce; 2) education and training; 3) midwifery-led service delivery, and 4) midwifery-led governance and leadership. Our findings provide important starting points to support midwives in Somaliland across all key areas. Notwithstanding the critical need for mental health support for midwives, especially those who have had traumatic experiences related to FGM/C.

Ethics statements

Patient consent for publication

Not applicable.

Ethics approval

This study involves human participants, and approval to conduct the study at the health facilities was obtained from the Somaliland Ministry of Health and Development MOHD/DG: 2/J-cG/2020. Ethical clearance was approved by the Ethical Board at the University of Hargeisa, Somaliland DRCS/28/5/2020.

Acknowledgments

We would like to thank the midwives who shared with us their personal lived experiences. Also, thank you Dalarna University for providing resources that necessitated this research.

Contributors

The basis for this study is a mutual long-term capacity building partnership between local and Swedish academic partners. The research process and the final manuscript follows The ICMJE recommended criteria for authorship. Conceptualization of the study design and method: RTM, KB, JAE, AE, CM, MKA. Planning and implementation of the study including data collection JAE, AE and RTM (online) supported by MKA. Data analysis was conducted by RTM, KB, JAE, CM supported by MKA. Data interpretation: RTM, KB, JAE, AE, CM, MKA. Writing: RTM, KB, CM, MKA. Editing and proofreading: RTM, KB, JAE, AE, CM, MKA.

Competing Interests

No conflict of interests has been declared.

For peer review only - http://bmjopen.bmj.com/site/about/guidelines.xhtml

Funding

This study was supported by Dalarna University with award Dnr N/A, Karolinska Institute with award Dnr N/A and Swedish Research Council with award Dnr 2020-04372. The funders played no part in the conduct of this research, and the views expressed in this publication are those of the authors.

Data availability statement

Data are available on reasonable request.

REFERENCES

1. Starrs A M, Ezeh A, Barker G, et al. Accelerate progress—sexual and reproductive health and rights for all: report of the Guttmacher–Lancet Commission. *Lancet* 2018;*391*(10140):2642–2692. https://doi.org/10.1016/S0140-6736(18)30293-9 [published Online First: 09 May 2018].
2. United Nations Population Fund. *Supplement to background paper on sexual and reproductive health and rights: an essential element of universal health coverage*, 2019. https://www.unfpa.org/featured-publication/sexual-and-reproductive-health-and-rights-essential-element-universal-health [Accessed 02/01/2021].
3. Renfrew M J, Ateva E, Dennis-Antwi A, et al. Midwifery is a vital solution—what is holding back global progress? *Birth* 2019;46(3):396–399. https://doi.org/10.1111/BIRT.12442 [published Online First: 03 July 2019]
4. United Nations. *Transforming our world: the 2030 Agenda for Sustainable Development | Department of Economic and Social Affairs*, 2015. https://sdgs.un.org/2030agenda [Accessed 12/03/2021].
5. United Nations Population Fund. *State of the World's Midwifery 2014*. Jan, 2014. https://www.unfpa.org/sowmy-2014 [Accessed 27/10/2021].
6. Nove A, Friberg I K, de Bernis L, et al. Potential impact of midwives in preventing and reducing maternal and neonatal mortality and stillbirths: a Lives Saved Tool modelling study. *Lancet Glob Health* 2020;9(1). https://doi.org/10.1016/s2214-109x(20)30397-1 [published Online First: 01 December 2020]
7. Beek K, McFadden A, Dawson A. The role and scope of practice of midwives in humanitarian settings: a systematic review and content analysis. *Hum Resour Health* 2019;17(1):5. https://doi.org/10.1186/s12960-018-0341-5 [published Online First: 14 January 2019]
8. Lukasse M, Pajalic Z. Norwegian midwives' perceptions of empowerment. *Sex Reprod Healthc* 2016;7:58–64. https://doi.org/10.1016/j.srhc.2015.11.010 [published Online First: 08 December 2015]
9. Mattison C A, Lavis J N, Wilson M G, et al. A critical interpretive synthesis of the roles of midwives in health systems. *Health Res Policy Syst* 2020;18(1):1–16. https://doi.org/10.1186/S12961-020-00590-0 [published Online First: 08 July 2020]

For peer review only

For peer review only - http://bmjopen.bmj.com/site/about/guidelines.xhtml

10. Central Statistics Department, Ministry of Planning and National Development, & Somaliland Government. *The Somaliland Health and Demographic Survey (SLHDS)*, 2020. www.somalilandmohd.com [Accessed 10/03/2021].

11. Kiruja J, Osman F, Egal J A, et al. Maternal near-miss and death incidences—frequencies, causes, and the referral chain in Somaliland: a pilot study using the WHO near-miss approach. *Sex Reprod Healthc* 2017;12:30–36. https://doi.org/10.1016/j.srhc.2017.02.003 [published Online First: 27 February 2017]

12. Egal, J. A., Kiruja, J., Litorp, H., et al. Incidence and causes of severe maternal outcomes in Somaliland using the sub-Saharan Africa maternal near-miss criteria: A prospective cross-sectional study in a national referral hospital. *Int. J. Gynecol. Obstet* 2022;00:1–9. https://doi.org/10.1002/IJGO.14236

13. Erlandsson K, Osman F, Hatakka M, et al. Evaluation of an online master's program in Somaliland. A phenomenographic study on the experience of professional and personal development among midwifery faculty. *Nurse Educ Pract* 2017; 25:96–103. https://doi.org/10.1016/J.NEPR.2017.05.007 [published Online First: 23 May 2017].

14. International Confederation of Midwifery. *Essential competencies for midwifery practice*, 2018. https://www.internationalmidwives.org/our-work/policy-and-practice/essential-competencies-for-midwifery-practice.html [Accessed 20/11/2020].

15. Garces A, McClure E M, Espinoza L, et al. Traditional birth attendants and birth outcomes in low-middle income countries: a review. *Semin Perinatol* 2019;43(5):247–251. https://doi.org/10.1053/J.SEMPERI.2019.03.013 [published Online First: 21 March 2019].

16. Robinson N. The use of focus group methodology—with selected examples from sexual health research. *J Adv Nurs* 1999;29(4):905–913. https://doi.org/10.1046/j.1365-2648.1999.00966.x [published Online First: 25 December 2001].

17. Tracy S J. Qualitative research methods: Collecting Evidence, Crafting Analysis, Communicating Impact. John Wiley & Sons Ltd 2020: 75-200.

18. Braun V and Clarke V, Using thematic analysis in psychology. *Qual Res Psychol 2006;*3(2):77–101. https://doi.org/10.1191/1478088706qp063oa [published Online First: 21 July 2008]

19. Mordal E, Hanssen I, Biratu A K, et al. Providing safe maternity care under challenging conditions in rural Ethiopia: a qualitative study. *BMC Health Serv Res* 2021;21(1):1–10. https://doi.org/10.1186/S12913-021-06324-4 [published Online First: 09 April 2021].

20. Feeley C, Thomson G, Downe S. Understanding how midwives employed by the National Health Service facilitate women's alternative birthing choices: findings from a feminist pragmatist study. *PLOS ONE* 2020;15(11):e0242508. https://doi.org/10.1371/journal.pone.0242508 [published Online First: 20 November 2020].

21. Ewnetu D B, Thorsen V C, Solbakk J H, et al. Still a moral dilemma: how Ethiopian professionals providing abortion come to terms with conflicting norms and demands. *BMC Med Ethics* 2020;21(1):1–7. https://doi.org/10.1186/S12910-020-0458-7 [published Online First: 11 February 2020].

For peer review only - http://bmjopen.bmj.com/site/about/guidelines.xhtml

22. Isman E, Mahmoud Warsame A, Johansson A, et al. Midwives' experiences in providing care and counselling to women with female genital mutilation (FGM) related problems. *Obstet Gynecol Int* 2013:1–9. https://doi.org/10.1155/2013/785148 [published Online First: 18 September 2013].

23. Leye E, van Eekert N, Shamu S, et al. Debating medicalization of female genital mutilation/cutting (FGM/C): learning from (policy) experiences across countries. *Reprod Health* 2019;16(1):1–10. https://doi.org/10.1186/S12978-019-0817-3 [published Online First: 01 November 2019].

24. Klingberg-Allvin, M., Cleeve, A., et al. Comparison of treatment of incomplete abortion with misoprostol by physicians and midwives at district level in Uganda: A randomized controlled equivalence trial. *The Lancet* 2015;385(9985):2392–2398. https://doi.org/10.1016/S0140-6736(14)61935-8 [published Online First: 26 March 2015].

25. Atuhairwe, S., Byamugisha, J., et al. (2019). Evaluating the safety, effectiveness and acceptability of treatment of incomplete second-trimester abortion using misoprostol provided by midwives compared with physicians: study protocol for a randomized controlled equivalence trial. *Trials 2019*;20(1). https://doi.org/10.1186/S13063-019-3490-5 [published Online First: 21 June 2019].

26. Somaliland Nursing and Midwifery Association. *Voices of nurses and midwives,* 2020. https://slnma.org/wp-content/uploads/2021/03/magazine-2.pdf [Accessed 14/10/2021].

27. Mattison, C., Bourret, K., Hebert, E., Leshabari, et al. Health systems factors impacting the integration of midwifery: an evidence-informed framework on strengthening midwifery associations. *BMJ Global Health* 2021;*6*(6). https://doi.org/10.1136/BMJGH-2020-004850 [published Online First: 21 January 2014].

28. Hailu DagneID, A., & Haile Beshah, M. Implementation of evidence-based practice*:* The experience of nurses and midwives. *PLoS One* 2021;16(8) : e0256600. https://doi.org/10.1371/journal.pone.0256600 [published Online First: 27 August 2021].

For peer review only - http://bmjopen.bmj.com/site/about/guidelines.xhtml

Reporting checklist for qualitative study.

Based on the SRQR guidelines.

Instructions to authors

Complete this checklist by entering the page numbers from your manuscript where readers will find each of the items listed below.

Your article may not currently address all the items on the checklist. Please modify your text to include the missing information. If you are certain that an item does not apply, please write "n/a" and provide a short explanation.

Upload your completed checklist as an extra file when you submit to a journal.

In your methods section, say that you used the SRQRreporting guidelines, and cite them as:

O'Brien BC, Harris IB, Beckman TJ, Reed DA, Cook DA. Standards for reporting qualitative research: a synthesis of recommendations. Acad Med. 2014;89(9):1245-1251.

		Reporting Item	Page Number
**Title**			
	#1	Concise description of the nature and topic of the study identifying the study as qualitative or indicating the approach (e.g. ethnography, grounded theory) or data collection methods (e.g. interview, focus group) is recommended	1
**Abstract**			
	#2	Summary of the key elements of the study using the abstract format of the intended publication; typically includes background, purpose, methods, results and conclusions	2
**Introduction**			
Problem formulation	#3	Description and signifcance of the problem / phenomenon studied: review of relevant theory and empirical work; problem statement	4-5

For peer review only

Purpose or research question	#4	Purpose of the study and specific objectives or questions	5
**Methods**			
Qualitative approach and research paradigm	#5	Qualitative approach (e.g. ethnography, grounded theory, case study, phenomenolgy, narrative research) and guiding theory if appropriate; identifying the research paradigm (e.g. postpositivist, constructivist / interpretivist) is also recommended; rationale. The rationale should briefly discuss the justification for choosing that theory, approach, method or technique rather than other options available; the assumptions and limitations implicit in those choices and how those choices influence study conclusions and transferability. As appropriate the rationale for several items might be discussed together.	5
Researcher characteristics and reflexivity	#6	Researchers' characteristics that may influence the research, including personal attributes, qualifications / experience, relationship with participants, assumptions and / or presuppositions; potential or actual interaction between researchers' characteristics and the research questions, approach, methods, results and / or transferability	5
Context	#7	Setting / site and salient contextual factors; rationale	5
Sampling strategy	#8	How and why research participants, documents, or events were selected; criteria for deciding when no further sampling was necessary (e.g. sampling saturation); rationale	5
Ethical issues pertaining to human subjects	#9	Documentation of approval by an appropriate ethics review board and participant consent, or explanation for lack thereof; other confidentiality and data security issues	5,15
Data collection methods	#10	Types of data collected; details of data collection procedures including (as appropriate) start and stop dates of data collection and analysis, iterative	5

For peer review only

		process, triangulation of sources / methods, and modification of procedures in response to evolving study findings; rationale	
Data collection instruments and technologies	#11	Description of instruments (e.g. interview guides, questionnaires) and devices (e.g. audio recorders) used for data collection; if / how the instruments(s) changed over the course of the study	5
Units of study	#12	Number and relevant characteristics of participants, documents, or events included in the study; level of participation (could be reported in results)	5,9
Data processing	#13	Methods for processing data prior to and during analysis, including transcription, data entry, data management and security, verification of data integrity, data coding, and anonymisation / deidentification of excerpts	5
Data analysis	#14	Process by which inferences, themes, etc. were identified and developed, including the researchers involved in data analysis; usually references a specific paradigm or approach; rationale	5,15
Techniques to enhance trustworthiness	#15	Techniques to enhance trustworthiness and credibility of data analysis (e.g. member checking, audit trail, triangulation); rationale	5
**Results/findings**			
Syntheses and interpretation	#16	Main findings (e.g. interpretations, inferences, and themes); might include development of a theory or model, or integration with prior research or theory	5-13
Links to empirical data	#17	Evidence (e.g. quotes, field notes, text excerpts, photographs) to substantiate analytic findings	5-13
**Discussion**			
Intergration with prior work, implications, transferability and contribution(s) to the field	#18	Short summary of main findings; explanation of how findings and conclusions connect to, support, elaborate on, or challenge conclusions of earlier scholarship; discussion of scope of application /	13-15

For peer review only

		generalizability; identification of unique contributions(s) to scholarship in a discipline or field	
Limitations	#19	Trustworthiness and limitations of findings	3,14
**Other**			
Conflicts of interest	#20	Potential sources of influence of perceived influence on study conduct and conclusions; how these were managed	15
Funding	#21	Sources of funding and other support; role of funders in data collection, interpretation and reporting	16

The SRQR checklist is distributed with permission of Wolters Kluwer © 2014 by the Association of American Medical Colleges. This checklist can be completed using https://www.goodreports.org/, a tool made by the EQUATOR Network in collaboration with Penelope.ai

For peer review only

BMJ Open

A qualitative study of the roles of midwives in the provision of sexual and reproductive health care services in the Somaliland health system.

Journal:	*BMJ Open*
Manuscript ID	bmjopen-2022-067315.R1
Article Type:	Original research
Date Submitted by the Author:	04-Feb-2023
Complete List of Authors:	Maregn, Rahel Tesfa; Dalarna University, School of Health and Welfare<br>Bourret, Kirsty; Karolinska Institute, Department of Women and Children’s Health; McMaster University, Department of Obstetrics and Gynecology<br>Egal, Jama Ali; Dalarna University, School of Health and Welfare; University of Hargeisa College of Medicine and Health Sciences, Faculty of Nursing and Midwifery<br>Esse, Amina; University of Hargeisa College of Medicine and Health Sciences, Faculty of Nursing and Midwifery<br>Mattison, Cristina; McMaster University, Department of Obstetrics and Gynecology; Karolinska Institute, Department of Women and Children’s Health<br>klingberg-allvin, marie; Dalarna University, School of Health and Welfare; Karolinska Institute, Department of Women and Children’s Health
**Primary Subject Heading**:	Health services research
Secondary Subject Heading:	Qualitative research, Nursing, Reproductive medicine, Sexual health, Public health
Keywords:	QUALITATIVE RESEARCH, PUBLIC HEALTH, Health Services Accessibility, Quality in health care < HEALTH SERVICES ADMINISTRATION & MANAGEMENT, Reproductive medicine < GYNAECOLOGY, SEXUAL MEDICINE

SCHOLARONE™
Manuscripts

I, the Submitting Author has the right to grant and does grant on behalf of all authors of the Work (as defined in the below author licence), an exclusive licence and/or a non-exclusive licence for contributions from authors who are: i) UK Crown employees; ii) where BMJ has agreed a CC-BY licence shall apply, and/or iii) in accordance with the terms applicable for US Federal Government officers or employees acting as part of their official duties; on a worldwide, perpetual, irrevocable, royalty-free basis to BMJ Publishing Group Ltd ("BMJ") its licensees and where the relevant Journal is co-owned by BMJ to the co-owners of the Journal, to publish the Work in this journal and any other BMJ products and to exploit all rights, as set out in our licence.

The Submitting Author accepts and understands that any supply made under these terms is made by BMJ to the Submitting Author unless you are acting as an employee on behalf of your employer or a postgraduate student of an affiliated institution which is paying any applicable article publishing charge ("APC") for Open Access articles. Where the Submitting Author wishes to make the Work available on an Open Access basis (and intends to pay the relevant APC), the terms of reuse of such Open Access shall be governed by a Creative Commons licence – details of these licences and which Creative Commons licence will apply to this Work are set out in our licence referred to above.

Other than as permitted in any relevant BMJ Author's Self Archiving Policies, I confirm this Work has not been accepted for publication elsewhere, is not being considered for publication elsewhere and does not duplicate material already published. I confirm all authors consent to publication of this Work and authorise the granting of this licence.

For peer review only

A qualitative study of the roles of midwives in the provision of sexual and reproductive health care services in the Somaliland health system

Rahel Tesfa Maregn [1]* Kirsty M. Bourret [2],[3], Jama Ali Egal [1],[4], Amina Esse [4], Cristina A. Mattison, PhD [2],[3], Marie Klingberg-Allvin [1],[2]*

[1] School of Health and Welfare,
Dalarna University, Falun, Sweden

[2] Department of Women and Children's Health,
Karolinska Institute, Stockholm, Sweden

[3] McMaster Midwifery Research Centre
Department of Obstetrics and Gynaecology
McMaster University, Toronto, Canada

[4] Faculty of Nursing and Midwifery
College of Medicine and Health Sciences
University of Hargeisa, Hargeisa, Somaliland

*Corresponding authors:
Rahel Tesfa Maregn, (MD)
School of Health and Welfare,
Dalarna University
791 88, Falun, Sweden
e-mail: h20rahem@du.se

Marie Klingberg-Allvin
Professor, Department of Women and Children's Health, Karolinska Institutet, Solna, Sweden.
School of Health and Welfare, Dalarna University, Faun, Sweden.
e-mail: marie.klingberg-allvin@ki.se
Cell phone: +46 707174414

Wordcount: 4382

For peer review only

ABSTRACT

Objectives: To explore midwives´ perspectives in providing sexual reproductive health care services in the Somaliland health system.

Methods: An exploratory qualitative design using focus group discussions (n = 6) was used. The study was conducted in the capital of Somaliland, Hargeisa, at six maternal and child health care centres that provide SRHR services. Qualified midwives (n=44) who had been working in the MCHs for a minimum of one year were recruited to participate, and only one did not participate due to illness

Results: The results showed that Somaliland midwives face multiple challenges from a lack of formal arrangements, primarily written guidelines and policies, that explicitly define their role as healthcare professionals, which impact the quality of care they provide. They also reported feeling unsafe when practising according to their professional scope of practice due to challenging cultural norms, customary traditions, and Somaliland's legal system. Finally, the midwives called for support, including training, institutional protection, and psychological support, to enhance their ability and fulfil their role in SRHR care in Somaliland.

Conclusion: Midwives are essential to the provision of equitable SRHR care to women and girls, yet are not fully supported by policies, laws, or institutions, often living in fear of the consequences of their behaviours. Our research highlights the importance of understanding the context of Somaliland midwifery in order to better support the development of the midwifery workforce, stronger governance structures and midwifery leadership. Appropriately addressing these challenges faced by midwives can better sustain the profession and help to improve the quality of care provided to women and girls and ultimately enhance their reproductive health outcomes.

Key terms: sexual reproductive health care, midwives, qualitative study, health systems research, Somaliland

For peer review only

STRENGTHS AND LIMITATIONS OF THIS STUDY

- Data were collected by two professional Somali researchers with midwifery backgrounds, coupled with a collaborative approach throughout the research process between the research team in and outside of Somaliland.
- The study participants were frontline workers with first-hand knowledge and experience of the occurrence of the phenomenon. These first-hand accounts provide rich insights into understanding the social and cultural context in which the provision of sexual and reproductive health services takes place.
- While there was much sharing during the focus group discussions, the setting may have limited how much individuals shared. Some may not have wanted to disclose personal details of their experiences in a group setting, especially given the sensitive topic areas.

For peer review only

For peer review only - http://bmjopen.bmj.com/site/about/guidelines.xhtml

Introduction

Sexual and reproductive health and rights (SRHR) interventions benefit women, girls, children, and societies at large. They are considered a cornerstone to achieving universal health coverage (UHC) and reaching the Sustainable Development Goals (SDGs).[1],[2] Following a life-course approach, the minimum standards of an essential package of SRHR care services include contraceptive counselling; induced abortion, post-abortion care (PAC), contraception, antenatal, intrapartum, and postnatal care; and detection and prevention of sexual and gender-based violence (SGBV).[2],[3] The provision of these essential SRHR care services would contribute to reducing the global maternal mortality ratio to fewer than 70 per 100,000 live births and attaining the target of universal access to SRHR, as set out in the 2030 Agenda for Sustainable Development.[4] Midwives who are educated and regulated according to international standards are an essential part of the health system and can meet 87% of the population's SRHR needs.[5] Midwife-led care, supported by an enabling environment, could save an estimated 4.3 million lives annually by 2035.[6] While scientific evidence shows that midwives are vital to the delivery of safe and effective SRHR care services, there are significant challenges in the provision of these services within and across countries due to social and cultural factors, gender inequalities and a lack of understanding of the role of midwifery in health systems.[3],[7]-[9]

Somaliland has a high annual maternal mortality rate, at an estimated 396 deaths per 100,000 live births.[10] The leading causes of maternal deaths are postpartum haemorrhage, pre-eclampsia/eclampsia, prolonged and obstructed labour, and infections combined with limited access to SRHR services.[10]-[12] The recent Somaliland Health and Demographic Report (2020) showed that 67% of children are delivered at home, further contributing to high maternal and neonatal mortality and morbidity. Moreover, cultural practices such as female genital mutilation/cutting (FGM/C) are prevalent. Approximately 98% of women and girls between the ages of 15–49 have undergone the procedure, 61% of whom underwent Pharaonic circumcision, a severe form of FGM/C. Access to contraceptives is limited, with 7% of married women reporting practising birth spacing, and only 1% use modern contraceptives.[10]

The limited availability of healthcare facilities and the extreme shortage of qualified healthcare professionals further impact health outcomes.[10],[11],[13] Despite these shortfalls, midwifery institutions and universities in Somaliland follow the International Confederation of Midwives (ICM) standards for midwifery education to meet global requirements and improve the quality of SRHR.[13],[14] There are 1313 midwives in Somaliland (993 with diploma certificates, 270 with Bachelor's degrees, and 50 with Master's degrees in midwifery) (Somaliland Nursing and Midwifery Association, 2021). Due to the shortage of healthcare professionals and midwives, low-resource settings like Somaliland often struggle with providing pregnant women with complete

For peer review only - http://bmjopen.bmj.com/site/about/guidelines.xhtml

health coverage. As a result, women living in these settings rely on non-professional traditional birth attendants who have acquired skills by assisting mothers during home-based childbirths without any medical training.[10]-[12],[15]

While there has been some research on the barriers to specific SRHR care services in Somaliland, to our knowledge, there is no research that explores the specific challenges and opportunities faced by midwives in this context. This includes understanding the barriers to providing SRHR care services, such as cultural and social norms, and the strategies midwives use to overcome these barriers. Therefore, our study aims to contribute to a better understanding of midwives' roles in providing SRHR care services in the Somaliland health system. By exploring the experiences and perspectives of midwives in this context, we can identify the challenges and opportunities they face and inform the development of policies and programs that support their efforts to provide essential SRHR care services. This paper is useful for policymakers, practitioners, and researchers working in SRHR in Somaliland and similar contexts, as the findings can inform future research and contribute to the development of evidence-based policies and programs to support and strengthen midwives´ capacity to provide SRHR care services to their full potential. These further improve access to and quality of SRHR care services and ultimately improve overall health outcomes.

Methods

We followed an exploratory qualitative study design and used focus group discussions (FGDs) with midwives for data collection.[16],[17] The data were analyzed inductively using thematic analysis.[18] Since this study is the first of its kind in Somaliland, this design allowed us to understand and describe the nature of the problem.

Data Collection

The study was conducted in the capital of Somaliland, Hargeisa, at six Maternal and Child Health Centres (MCHs). MCHs are public facilities monitored by the Ministry of Health. MCHs will either receive clients directly or if they are referred from primary health units, and they must provide basic emergency obstetric and newborn care (EmONC). The MCHs should be staffed by at least one midwife, a nurse, an auxiliary nurse, and a community midwife and cover a population of approximately 5000 people. Six MCHs were targeted for data collection because they served displaced people and host communities, were of equal size, and the care was mainly provided by midwives.

A purposive sampling approach was used to recruit practising midwives from the six MCHs. Qualified midwives who met the inclusion criteria had been working in the MCHs for a minimum of one year and had experience with SRHR care service provision. A total of 44 midwives were recruited to participate via face-to-face invitations, and only one did not show up due to illness. The participants were given oral and written information about the study before consent. Six FGDs were conducted in the respective MCHs between January and February 2021. The research team

For peer review only - http://bmjopen.bmj.com/site/about/guidelines.xhtml

designed an FGD topic guide to facilitate discussion and aid in data collection (See Appendix 1).[16],[17] The guide was piloted in one of the MCHs with seven participants, after which a few probing questions were added. All the FGDs were facilitated by JAE and assisted by AE both with midwifery backgrounds. Except for one FGD that was conducted in English (RTM was present online), all were conducted in person in the Somali language. Each FGD took approximately one and a half hours.

Analysis

The audio recordings were transcribed and translated into English by a Somali translator. The transcribed data were checked for accuracy (JAE) and de-identified. In the first step, the entire team carefully read the transcripts of the FGDs multiple times to immerse and become completely familiar with them. The data were then inserted into NVivo-12, where the analysis team (RTM, KB, and CM) coded the data using a thematic analysis approach using Braun and Clarke.[18] Codes were reviewed and discussed with the research team (JAE, AE, and MKA). Based on patterns and commonalities, similar codes were grouped as sub-themes and themes (RTM, KB, and CM) and subsequently reviewed and revised multiple times until the research group reached an agreement that reflected the content of the data (See Table 1). Feedback was obtained from the participants after sharing the results (AE, JAE).

Table 1: Condensed meaning units and generic codes showing the data extraction process.

Themes	Subthemes	Generic codes
**Somaliland context and its impact on midwives**	*Sustaining SRHR care services in the face of resource constraints*	Service improvisation with available resources<br>Fundraising<br>Counselling and referral<br>Lack of midwives<br>Lack of service guideline<br>Need for in-service training<br>Service provision in unsafe working environments<br>Need for occupational support

For peer review only - http://bmjopen.bmj.com/site/about/guidelines.xhtml

	Professional, cultural, and legal dilemmas	Husbands' consent for family planning and post-abortion care<br>Unsafe abortion practices<br>Acting outside the scope of practice<br>Treats and pressures from the client´s family members<br>Pregnancy outside marriage and stigma<br>Sociocultural norms behind FGM/C<br>Psychological traumas related to lived experiences of FGM/C<br>Families dealing with sexual violence internally
**Midwives' perceptions of their role in broader SRHR**	*Provision of family planning and post-abortion care*	Responsible for providing family planning and PAC<br>Premedical assessment<br>Counselling and couples' mediation,<br>Breastfeeding guidance as a way of child spacing<br>Referrals and liaising
	FGM/C and sexual and gender-based violence	Treating post-FGM/C complications<br>Educating clients about FGM/C<br>Community awareness<br>Using opportunities<br>Providing care for survivors of sexual and gender-based violence<br>Providing care to women with pregnancy outside of marriage
	Midwives as advocates and system navigators for clients	Being a role model in using services<br>Use of religious scripts to support women<br>Use female and male support groups for community reach out<br>Women's educational and empowerment programs

Patient and public involvement
None.

Results
In the analysis, two main themes were identified: 1) the Somaliland context and its impact on midwives (i.e., midwives' working environment at the intersection of health, sociocultural and legal systems), and 2) midwives' perceptions of their role in broader SRHR in the context of Somaliland. Each of these included related sub-themes. Context-specific terminologies, such as

For peer review only
For peer review only - http://bmjopen.bmj.com/site/about/guidelines.xhtml

child spacing/birth spacing (defined as family planning or contraceptive use) and illegal pregnancy (means the pregnancy has been conceived outside of marriage), were used. The following sub-themes elaborate on how the midwives described the lack of occupational and legal support in their work environments and the tensions they experienced between sociocultural norms and their professional role.

Somaliland context and its impact on midwives

In this theme, midwives describe how the health, sociocultural and legal systems in Somaliland impacted them while providing sexual reproductive health and rights services.

Sustaining SRHR care services in the face of resource constraints

The midwives stated that there are frequent disruptions in medical equipment and pharmacological supplies and a lack of emergency transport.

> *We give women all the family planning information, unfortunately, we do not provide family planning in this MCH. Those who need family planning, we connect/transfer to other MCHs*. (Focus Group 1)

A lack of contraceptives was seen as particularly detrimental, leaving many women exposed to the risk of unintended pregnancy. Furthermore, a lack of supplies meant that the midwives could not manage emergencies, and a lack of transport meant that instead of treating women, they became logisticians, using their cars or collecting funds for taxi fees to escort women to referral hospitals.

> *We don't have a scanner in the MCH, so we have no way of assessing the level of care a woman needs here […]. If a woman is bleeding severely, we ensure that there is access to a cannula, and we transfer them to the hospital using the team leader's personal car, as the MCH has no ambulance, which we really need.* (Focus Group 5)

According to the participants, challenges in service delivery arrangements, such as unclear interprofessional roles and responsibilities, high workloads due to a shortage of midwives, and overcrowded hospitals, severely limit the quality of care and contribute to adverse outcomes.

> *There is a shortage of midwives in Somaliland hospitals […] they have a lot of tasks that occupy their time, overcrowding of people in the hospitals[…] Midwives don't make enough money to sustain their lives, so a lot of them are working double jobs and shifts. There is no monitoring or evaluation from the Ministry as well.* (Focus group 6)

The participants claimed that the lack of access to training and hands-on supervision impacted their confidence in providing SRHR service to their clients within the scope of their professional duties. For example, not all midwives have been trained in post-abortion care or feel equipped to implement life-saving skills when faced with complications from incomplete abortions.

For peer review only - http://bmjopen.bmj.com/site/about/guidelines.xhtml

> *We feel it is our job as midwives to help save mothers when they experience early pregnancy complications, but it is sad we cannot.* (Focus Group 5)

> *We, midwives, need more training and experience. We would have a lot of impacts. (*Focus Group 6)

They also mentioned a lack of care guidelines, definitions of tasks, and legal and occupational support.

> *There is no legal framework [concerning family planning services] in Somaliland. This lack means healthcare workers do not get the support they need to support women.* (Focus Group 5)

Professional, cultural, and legal dilemmas

The participants described how the blending of boundaries between cultural norms, customary traditions, and Somaliland's legal system shapes their decision-making and, ultimately, the care pathways of their clients. In Somaliland, there are currently no laws either allowing or prohibiting women from consenting to their own care management. However, cultural norms dictate that either a husband or a male family member must consent to the care, even in urgent or life-threatening circumstances such as an incomplete abortion.

> *When a woman comes in with abortion complications, you can't help her without her husband. You need the husband present because you need him to sign the consent form, so you don't face any problems afterwards because anything is possible.* (Focus Group 6)

> *Some husbands would, without a doubt, come to us carrying a gun if they ever found out we gave their wives contraceptives without their consent [...] but legally, there is no law against giving any method to a woman.* (Focus Group 3)

The midwives felt they were professionally and ethically required to provide services to their clients, yet both the midwife and the client could face serious consequences if they did not seek male consent. For example, contrary to cultural norms, it is within the regulatory framework to provide contraception to women without male consent. However, midwives reported being put in unsafe positions if they did so. Verbal, physical, psychological, and legal threats from clients' family members were common occurrences in their day-to-day practices.

> *In the morning, the husband came into the MCHs with a big wooden stick and said, 'Bring out the women in the staff; she will die, or I will.' The police were called, and the husband told the police personnel that the midwife had stopped the childbearing of his wife. There is no law that can guarantee or protect healthcare staff from issues like these.* (Focus Group 1)

For peer review only - http://bmjopen.bmj.com/site/about/guidelines.xhtml

Unfortunately, we as health care professionals have no law to protect us against a family dispute or if the husband is unhappy about the decision. (Focus Group 3)

These discussions were the most prominent regarding the provision of family planning, emergency post-abortion care, and supporting survivors of SGBV.

FGM/C is common practice in Somaliland, and participants linked it to sociocultural beliefs around gender norms that include purity, prevention of pregnancy outside marriage and marriage acceptance.

The women believe that all girls who had been performed Sunni (in comparison to the Pharaonic type) are doing an illegal sexual activity, and they are doing naughty things that are illegal […]We (midwives) met many girls who had been circumcised and they still have illegal pregnancy. (Focus Group 2)

Most mothers believe that their daughters will not be accepted for marriage if they don't have a Pharaonic circumcision. This also includes most men. They say the girl is not pure. (Focus Group 5)

Woven into their narratives were how the midwives were psychologically and emotionally impacted by their own experiences with FGM/C when caring for women who had undergone FGM/C, which is a common occurrence given the extent of the practice.

The woman who cut me really traumatized me. Even years after being cut, I remember getting goosebumps all over my body whenever I saw her. I had ten people holding me down while I was being cut. Now that I know better, I can say what she did to me was completely destructive and damaging. Even to this day, it bothers me to see someone in pain during childbirth. I was basically someone who got surgery without anaesthesia. I was being cut up, and it is still very traumatic. (Focus Group 3)

In terms of psychological distress, all midwives in Somaliland are women, of which most have undergone FGM/C. Participants said some midwives continue to suffer trauma from their own lived experiences with FGM/C. They described how some midwives experience recurring traumatization in their day-to-day work through treating and supporting women and young girls with complications of FGM/C. They also described a lack of psychological support after providing services to these women and girls. The midwives highlighted the importance of receiving mental health support to better care for themselves and their clients.

We, as midwives, need psychological support to deal with our own trauma; only then can we support women. (Focus Group 3)

For peer review only - http://bmjopen.bmj.com/site/about/guidelines.xhtml

Midwives described how sociocultural expectations regarding FGM/C led to ethically difficult scenarios, which at times led to acting outside of their scope of practice. The midwives viewed these instances as a harm-reduction approach to the complications and long-term effects of FGM/C. The midwives also claimed that there is a shift in the demand for FGM/C services from community circumcisers to MCHs, and there is mounting social pressure on midwives to provide these services. Often, families cite the risks of turning to unsafe community circumcisers to coerce midwives into providing FGM/C.

> *A woman said, 'I need circumcisions […] If you refuse, I will get someone else to come in and do it for me.' I refused, and she tried to give me money to do that.* (Focus Group 1)

The midwives recognized that refusing to provide FGM/C would inevitably result in them having to treat girls with complications after they were taken to community circumcisers. The risk to the girls, combined with the midwives´ own trauma, compelled them to act, even going as far as performing Sunni circumcisions (described by the focus groups as cutting off the tip of the clitoris). They comply as a way of protecting the girls from a more severe form of FGM/C and related complications.

> *The reason they perform the Sunni type is to prevent girls from undergoing the Pharaonic type. (*Focus group 2)

On the other hand, midwives expressed feeling that the community saw them as playing contradictory roles by performing FGM/C yet promoting its decline.

> *How is it possible to encourage the community to stop FGM and, on the other hand, we continue the Sunni type?* (Focus Group 2)

In the discussions, some midwives questioned the cultural norms behind FGM/C and its practice in the MCHs.

> *Where does this idea of making a girl 'halaal' (pure) even come from? [...] Well, if midwives are given rules to follow, they all need to obey those rules. So, rules need to be created. Holding midwives accountable and having consequences for their actions will make them listen.* **(**Focus Group 3)

Midwives in all six focus groups discussed how the complex intersections of their personal experiences, professional roles, cultural norms, and legal framework impacted their personal and professional roles and their capacity to provide quality care to women and girls

Midwives' perceptions of their role in broader SRHR

For peer review only - http://bmjopen.bmj.com/site/about/guidelines.xhtml

When midwives were asked about post-abortion care and contraception, discussions organically led to FGM/C, sexual and gender-based violence, and how they advocated for women's SRHR. The three subthemes are discussed below.

Provision of post-abortion care and family planning services

The midwives said that they remained as the primary care providers for women needing post-abortion care and family planning.

> *Family planning is part of our services, and we tell every mother that comes to the health facility about child spacing*. (Focus Group 2)

> *Midwives have the main responsibility as we see women in pregnancy, labour, and postnatally*. (Focus Group 3)

The extent of family planning services provided by midwives includes premedical assessment, family planning counselling, couples' mediation, nutritional support, breastfeeding guidance, and liaising and referrals based on women's needs. The midwives provided family planning methods if they were available. While most participants mentioned that it is their responsibility to provide post-abortion care, only in one of the FGDs did the participants mention their competence and willingness to provide post-abortion care.

> *We do post-abortion care; we got training[…]we use misoprostol*. (Focus Group 1)

FGM/C and sexual and gender-based violence

As mentioned in the previous theme, midwives discussed how the practice of FGM/C impacted their roles as both midwives and women. They stated that their role included providing treatment for emergency and long-term complications of FGM/C, which includes treatment of post-FGM/C bleeding, infection, routine intrapartum care, psychological support, and SRHR education relating to FGM/C.

> *As a midwife, I tell my clients the best way to prevent illegal behaviour is to educate your children in a proper way*. (Focus Group 2)

The midwives supported reducing the FGM/C practice overall through community awareness and educating mothers about the cultural misconceptions and consequences of FGM/C. They explained that their role as educators and awareness creators brought them satisfaction, especially with regard to their personal experience with FGM/C.

> *When we have mothers come to us about other issues, like breastfeeding, we use these opportunities to educate them about the issues and problems FGM can cause*. (Focus Group 3)

For peer review only - http://bmjopen.bmj.com/site/about/guidelines.xhtml

Midwives provide frontline care to survivors of sexual and gender-based violence and women who get pregnant outside of marriage. The participants said they provided equal services without prejudice and gave examples of counselling women and girls who got pregnant outside of marriage.

> *Every week we come across two to three cases of rape, and most of those are kids being raped by family members[…] All women have the same options, regardless of where they come from. (*Focus Group 6)

<u>Midwives as advocates and system navigators for clients</u>

The midwives' gender intersected with their professional roles, and some participants mentioned seeing themselves as role models and using their status to support other women. For example, they highlighted the benefits of SRHR services to women and encouraged them to become role models in their respective communities. They also believed that midwives' uptake of services could impact social norms and society, in general, to be more accepting and open to change.

> *We all used it and advised each other about it (family planning*). (Focus Group 4)

> *After the training is finished, they (fathers and mothers' groups) go around villages and look for people with problems. They work with us now and are so valuable and powerful in the community.* (Focus Group 1)

In addition to supporting women and each other professionally, they used religious scripts and involved trusted community members in forming male and female support groups, which proved useful for community reach out and social change.

> *We are lucky that we have religious evidence that encourages family spacing […] We always use this to our advantage to support mothers.* (Focus Group 5)

In some cases, the midwives mentioned acting as advocates for rape survivors and supporting victims to prosecute their perpetrators.

> *We asked for an investigation and requested laws and regulations. In this specific case, we ended up winning, getting everyone off our backs, and finally sentencing the guy to jail for 20 years***. (**Focus Group 6)

The midwives also created programs to teach women about their reproductive rights and create women´s health and social awareness within their communities. This awareness strategy was used to empower women and increase their access to SRHR care services.

For peer review only - http://bmjopen.bmj.com/site/about/guidelines.xhtml

Discussion

To our knowledge, this is the first study to explore the role of midwives in the provision of SRHR in Somaliland and from the perspectives of midwives. Participants described their role expanding beyond antenatal and delivery care to primarily include PAC, contraceptive counselling, SGBV, FGM/C, and counselling concerning unintended pregnancies (i.e., pregnancies out of wedlock). They also described how they navigate legal, cultural, and social spheres, finding creative ways to mitigate and circumvent obstacles for women and girls in dire circumstances. Somaliland midwives face multiple challenges from a lack of formal arrangements, primarily written guidelines and policies, that explicitly define their role as healthcare professionals, leaving them vulnerable to arbitrary persecution and other social consequences. They felt a lack of clinical guidance when managing intrapartum care for women with FGM/C. Furthermore, their lived experiences as women and survivors of FGM/C caused them to experience trauma and suffering in their day-to-day life with little or no mental health support. Under extreme mental stress and lack of safety, midwives are in a constant dilemma. Their decision-making process intersects with their professional, community, religious, and individual selves.

Similar studies from different contexts have also shown how midwives improvise strategies to provide emergency SRHR care when there is a lack of resources.[19],[20] In the absence of clear guidelines and policies, healthcare professionals tend to employ their individual interpretations of social norms and laws.[21] Our results showed no consensus regarding appropriate strategies to address FGM/C practices. Some midwives attempted to deter communities from this sociocultural norm by citing the sexual and reproductive health risks it poses to women and girls. Others argued that FGM/C should be performed in legal facilities to reduce adverse outcomes of unsafe procedures conducted by non-medical individuals outside clinical settings. Some felt coerced and forced to agree to families' requests. Similar studies in Somaliland and elsewhere have also highlighted the controversies surrounding FGM/C, showing a lack of reconciliation between the two sides of the FGM/C practice spectrum.[22],[23]

Our results provide further insight into how midwives' experiences of working with cases of FGM/C and their lived experiences in the Somaliland community may come into play, causing distress and influencing clinical decision-making. This includes navigating the system to provide locally stigmatized SRHR services such as PAC, services to women with out-of-wedlock pregnancies, and support survivors of SGBV. Midwifery-led interventions, such as contraceptive counselling, are associated with the efficient use of resources and a positive impact on women's reproductive health outcomes.[6] Recent scientific evidence from similar settings shows midwives could play an essential role in PAC treating first and second-trimester incomplete abortion using misoprostol.[24],[25] Reports from Somaliland have highlighted that to increase women's access to evidence-based SRHR, midwives must be supported within the health system to broaden their scope of practice in correspondence to ICM Global Standards. The 2020 Somaliland Nursing and Midwifery Association report posits that midwives contribute to improving overall population

health and empowering individuals and communities.[26] Midwives in our study clearly state that to continue their work, they require the resources to manage the complexity of the experiences women and girls in Somaliland face regularly. They asked that they urgently receive trauma-informed mental health care so they can provide trauma-informed care to women, girls, and their communities. While our results highlight the positive impact of midwifery-led care for women and girls, they demonstrate the urgent need to invest in structural support for the profession.

The main strength of our study is the richness of the data, which was collected by two professional Somali women with midwifery backgrounds, coupled with a collaborative approach throughout the research process between the research team in and outside of Somaliland. Research generated by midwives is needed to create evidence-informed policies to support the integration of the profession into health systems.[9],[27],[28] While there was much sharing during the FDGs, the setting may have limited how much individuals shared, as some may not have wanted to disclose personal details of their experiences in a group setting, especially given the sensitive topic areas.

Conclusion

Maternal mortality and morbidity remain a significant challenge in Somaliland, especially in relation to services that are highly stigmatized, such as contraception, abortion care (including post-abortion care), and sexual and gender-based violence. Midwives are trusted members of the community and are often the first point of care for such services. They truly are integral to providing women and girls with safe, equitable SRHR. Our research provides a rich portrayal of midwives who are essential to the provision of equitable SRHR, yet are not fully supported by policies, laws, or institutions, often living in fear of the consequences of their behaviours. Our research highlights the importance of 1) the development and implementation of context-based written guidelines and policies that clearly define the role of midwives and provide them with clinical guidance, 2) investing in the professional development of midwives by providing training and supporting their efforts to provide SRHR services through the provision of necessary resources and equipment in Somaliland and similar settings, and 3) the critical need for mental health support for midwives, especially those who have had traumatic experiences related to FGM/C. By addressing the challenges and controversies facing midwives, we can help to improve the quality of care provided to women and girls and ultimately enhance their reproductive health outcomes.

Ethics statements

Patient consent for publication

Not applicable.

Ethics approval

This study involves human participants, and approval to conduct the study at the health facilities was obtained from the Somaliland Ministry of Health and Development MOHD/DG: 2/J-cG/2020.

For peer review only - http://bmjopen.bmj.com/site/about/guidelines.xhtml

Ethical clearance was approved by the Ethical Board at the University of Hargeisa, Somaliland DRCS/28/5/2020.

Acknowledgements

We would like to thank the midwives who shared with us their personal lived experiences. Also, thank you, Dalarna University, for providing the resources that necessitated this research.

Contributors

The basis for this study is a mutual long-term capacity-building partnership between local and Swedish academic partners. The research process and the final manuscript follow The ICMJE-recommended criteria for authorship. Conceptualization of the study design and method: RTM, KB, JAE, AE, CM, MKA. Planning and implementation of the study, including data collection JAE, AE, and RTM (online) supported by MKA. Data analysis was conducted by RTM, KB, JAE, and CM supported by MKA. Data interpretation: RTM, KB, JAE, AE, CM, MKA. Writing: RTM, KB, CM, MKA. Editing and proofreading: RTM, KB, JAE, AE, CM, MKA.

Competing Interests

No conflict of interest has been declared.

Funding

This study was supported by Dalarna University with an award of Dnr N/A, Karolinska Institute with an award Dnr N/A, and Swedish Research Council with an award Dnr 2020-04372. The funders played no part in the conduct of this research, and the views expressed in this publication are those of the authors.

Data availability statement

Data are available on reasonable request.

REFERENCES

1. Starrs A M, Ezeh A, Barker G, et al. Accelerate progress—sexual and reproductive health and rights for all: report of the Guttmacher–Lancet Commission. *Lancet* 2018;*391*(10140):2642–2692. https://doi.org/10.1016/S0140-6736(18)30293-9 [published Online First: 09 May 2018].
2. United Nations Population Fund. *Supplement to a background paper on sexual and reproductive health and rights: an essential element of universal health coverage*, 2019. https://www.unfpa.org/featured-publication/sexual-and-reproductive-health-and-rights-essential-element-universal-health [Accessed 02/01/2021].
3. Renfrew M J, Ateva E, Dennis-Antwi A, et al. midwifery is a vital solution—what is holding back global progress? *Birth* 2019;46(3):396–399. https://doi.org/10.1111/BIRT.12442 [published Online First: 03 July 2019]

For peer review only - http://bmjopen.bmj.com/site/about/guidelines.xhtml

4. United Nations. *Transforming our world: the 2030 Agenda for Sustainable Development | Department of Economic and Social Affairs*, 2015. https://sdgs.un.org/2030agenda [Accessed 12/03/2021].

5. United Nations Population Fund. *State of the World's Midwifery 2014*. Jan, 2014. https://www.unfpa.org/sowmy-2014 [Accessed 27/10/2021].

6. Nove A, Friberg I K, de Bernis L, et al. Potential impact of midwives in preventing and reducing maternal and neonatal mortality and stillbirths: a Lives Saved Tool modelling study. *Lancet Glob Health* 2020;9(1). https://doi.org/10.1016/s2214-109x(20)30397-1 [published Online First: 01 December 2020]

7. Beek K, McFadden A, Dawson A. The role and scope of practice of midwives in humanitarian settings: a systematic review and content analysis. *Hum Resour Health* 2019;17(1):5. https://doi.org/10.1186/s12960-018-0341-5 [published Online First: 14 January 2019]

8. Lukasse M, Pajalic Z. Norwegian midwives' perceptions of empowerment. *Sex Reprod Healthc* 2016;7:58–64. https://doi.org/10.1016/j.srhc.2015.11.010 [published Online First: 08 December 2015]

9. Mattison C A, Lavis J N, Wilson M G, et al. A critical interpretive synthesis of the roles of midwives in health systems. *Health Res Policy Syst* 2020;18(1):1–16. https://doi.org/10.1186/S12961-020-00590-0 [published Online First: 08 July 2020]

10. Central Statistics Department, Ministry of Planning and National Development, & Somaliland Government. *The Somaliland Health and Demographic Survey (SLHDS)*, 2020. www.somalilandmohd.com [Accessed 10/03/2021].

11. Kiruja J, Osman F, Egal J A, et al. Maternal near-miss and death incidences—frequencies, causes, and the referral chain in Somaliland: a pilot study using the WHO near-miss approach. *Sex Reprod Healthc* 2017;12:30–36. https://doi.org/10.1016/j.srhc.2017.02.003 [published Online First: 27 February 2017]

12. Egal, J. A., Kiruja, J., Litorp, H., et al. Incidence and causes of severe maternal outcomes in Somaliland using the sub-Saharan Africa maternal near-miss criteria: A prospective cross-sectional study in a national referral hospital. *Int. J. Gynecol. Obstet* 2022;00:1–9. https://doi.org/10.1002/IJGO.14236

13. Erlandsson K, Osman F, Hatakka M, et al. Evaluation of an online master's program in Somaliland. A phenomenographic study on the experience of professional and personal development among midwifery faculty. *Nurse Educ Pract* 2017; 25:96–103. https://doi.org/10.1016/J.NEPR.2017.05.007 [published Online First: 23 May 2017].

14. International Confederation of Midwifery. *Essential competencies for midwifery practice*, 2018. https://www.internationalmidwives.org/our-work/policy-and-practice/essential-competencies-for-midwifery-practice.html [Accessed 20/11/2020].

15. Garces A, McClure E M, Espinoza L, et al. Traditional birth attendants and birth outcomes in low-middle income countries: a review. *Semin Perinatol* 2019;43(5):247–251. https://doi.org/10.1053/J.SEMPERI.2019.03.013 [published Online First: 21 March 2019].

For peer review only

For peer review only - http://bmjopen.bmj.com/site/about/guidelines.xhtml

16. Robinson N. The use of focus group methodology—with selected examples from sexual health research. *J Adv Nurs* 1999;29(4):905–913. https://doi.org/10.1046/j.1365-2648.1999.00966.x [published Online First: 25 December 2001].

17. Tracy S J. Qualitative research methods: Collecting Evidence, Crafting Analysis, Communicating impact. John Wiley & Sons Ltd 2020: 75-200.

18. Braun V and Clarke V, Using thematic analysis in psychology. *Qual Res Psychol 2006;*3(2):77–101. https://doi.org/10.1191/1478088706qp063oa [published Online First: 21 July 2008]

19. Mordal E, Hanssen I, Biratu A K, et al. Providing safe maternity care under challenging conditions in rural Ethiopia: a qualitative study. *BMC Health Serv Res* 2021;21(1):1–10. https://doi.org/10.1186/S12913-021-06324-4 [published Online First: 09 April 2021].

20. Feeley C, Thomson G, Downe S. Understanding how midwives employed by the National Health Service facilitate women's alternative birthing choices: findings from a feminist pragmatist study. *PLOS ONE* 2020;15(11):e0242508. https://doi.org/10.1371/journal.pone.0242508 [published Online First: 20 November 2020].

21. Ewnetu D B, Thorsen V C, Solbakk J H, et al. Still a moral dilemma: how Ethiopian professionals providing abortion come to terms with conflicting norms and demands. *BMC Med Ethics* 2020;21(1):1–7. https://doi.org/10.1186/S12910-020-0458-7 [published Online First: 11 February 2020].

22. Isman E, Mahmoud Warsame A, Johansson A, et al. Midwives' experiences in providing care and counselling to women with female genital mutilation (FGM) related problems. *Obstet Gynecol Int* 2013:1–9. https://doi.org/10.1155/2013/785148 [published Online First: 18 September 2013].

23. Leye E, van Eekert N, Shamu S, et al. Debating medicalization of female genital mutilation/cutting (FGM/C): learning from (policy) experiences across countries. *Reprod Health* 2019;16(1):1–10. https://doi.org/10.1186/S12978-019-0817-3 [published Online First: 01 November 2019].

24. Klingberg-Allvin, M., Cleeve, A., et al. comparison of treatment of incomplete abortion with misoprostol by physicians and midwives at district level in Uganda: A randomized controlled equivalence trial. *The Lancet* 2015;385(9985):2392–2398. https://doi.org/10.1016/S0140-6736(14)61935-8 [published Online First: 26 March 2015].

25. Atuhairwe, S., Byamugisha, J., et al. (2019). Evaluating the safety, effectiveness and acceptability of treatment of incomplete second-trimester abortion using misoprostol provided by midwives compared with physicians: study protocol for a randomized controlled equivalence trial. *Trials 2019*;20(1). https://doi.org/10.1186/S13063-019-3490-5 [published Online First: 21 June 2019].

26. Somaliland Nursing and Midwifery Association. *Voices of nurses and midwives,* 2020. https://slnma.org/wp-content/uploads/2021/03/magazine-2.pdf [Accessed 14/10/2021].

27. Mattison, C., Bourret, K., Hebert, E., Leshabari, et al. Health systems factors impacting the integration of midwifery: an evidence-informed framework on strengthening midwifery associations. *BMJ Global Health* 2021;*6*(6). https://doi.org/10.1136/BMJGH-2020-004850 [published Online First: 21 January 2014].

28. Hailu DagneID, A., & Haile Beshah, M. Implementation of evidence-based practice*:* The experience of nurses and midwives. *PLoS One* 2021;16(8) : e0256600. https://doi.org/10.1371/journal.pone.0256600 [published Online First: 27 August 2021].

For peer review only - http://bmjopen.bmj.com/site/about/guidelines.xhtml

Appendix 1

Focus Group Discussion guide

1. What are your views and perceptions as a midwife in terms of your role in family planning (FP) and Post Abortion Care (PAC)?

2. What are the responsibilities and roles of midwives in relation to FP and PAC?

3. What are the challenges you face in providing these services (FP and PAC)?
   - What are the legal and socio-cultural frameworks for providing these services in your community?
   - In what ways do these frameworks impact you in your role as a midwife?

4. What support or resources do you feel would be most helpful for you to better fulfill your roles?

5. Is there anything else you would like to add or discuss related to your experiences as a midwife in Somaliland?

For peer review only

Reporting checklist for qualitative study.

Based on the SRQR guidelines.

Instructions to authors

Complete this checklist by entering the page numbers from your manuscript where readers will find each of the items listed below.

Your article may not currently address all the items on the checklist. Please modify your text to include the missing information. If you are certain that an item does not apply, please write "n/a" and provide a short explanation.

Upload your completed checklist as an extra file when you submit to a journal.

In your methods section, say that you used the SRQRreporting guidelines, and cite them as:

O'Brien BC, Harris IB, Beckman TJ, Reed DA, Cook DA. Standards for reporting qualitative research: a synthesis of recommendations. Acad Med. 2014;89(9):1245-1251.

		Reporting Item	Page Number
**Title**			
	#1	Concise description of the nature and topic of the study identifying the study as qualitative or indicating the approach (e.g. ethnography, grounded theory) or data collection methods (e.g. interview, focus group) is recommended	1
**Abstract**			
	#2	Summary of the key elements of the study using the abstract format of the intended publication; typically includes background, purpose, methods, results and conclusions	2
**Introduction**			
Problem formulation	#3	Description and signifcance of the problem / phenomenon studied: review of relevant theory and empirical work; problem statement	4-5

For peer review only

Purpose or research question	#4	Purpose of the study and specific objectives or questions	5
**Methods**			
Qualitative approach and research paradigm	#5	Qualitative approach (e.g. ethnography, grounded theory, case study, phenomenolgy, narrative research) and guiding theory if appropriate; identifying the research paradigm (e.g. postpositivist, constructivist / interpretivist) is also recommended; rationale. The rationale should briefly discuss the justification for choosing that theory, approach, method or technique rather than other options available; the assumptions and limitations implicit in those choices and how those choices influence study conclusions and transferability. As appropriate the rationale for several items might be discussed together.	5
Researcher characteristics and reflexivity	#6	Researchers' characteristics that may influence the research, including personal attributes, qualifications / experience, relationship with participants, assumptions and / or presuppositions; potential or actual interaction between researchers' characteristics and the research questions, approach, methods, results and / or transferability	5
Context	#7	Setting / site and salient contextual factors; rationale	5
Sampling strategy	#8	How and why research participants, documents, or events were selected; criteria for deciding when no further sampling was necessary (e.g. sampling saturation); rationale	5
Ethical issues pertaining to human subjects	#9	Documentation of approval by an appropriate ethics review board and participant consent, or explanation for lack thereof; other confidentiality and data security issues	5,15
Data collection methods	#10	Types of data collected; details of data collection procedures including (as appropriate) start and stop dates of data collection and analysis, iterative	5

For peer review only

		process, triangulation of sources / methods, and modification of procedures in response to evolving study findings; rationale	
Data collection instruments and technologies	#11	Description of instruments (e.g. interview guides, questionnaires) and devices (e.g. audio recorders) used for data collection; if / how the instruments(s) changed over the course of the study	5
Units of study	#12	Number and relevant characteristics of participants, documents, or events included in the study; level of participation (could be reported in results)	5,9
Data processing	#13	Methods for processing data prior to and during analysis, including transcription, data entry, data management and security, verification of data integrity, data coding, and anonymisation / deidentification of excerpts	5
Data analysis	#14	Process by which inferences, themes, etc. were identified and developed, including the researchers involved in data analysis; usually references a specific paradigm or approach; rationale	5,15
Techniques to enhance trustworthiness	#15	Techniques to enhance trustworthiness and credibility of data analysis (e.g. member checking, audit trail, triangulation); rationale	5
**Results/findings**			
Syntheses and interpretation	#16	Main findings (e.g. interpretations, inferences, and themes); might include development of a theory or model, or integration with prior research or theory	5-13
Links to empirical data	#17	Evidence (e.g. quotes, field notes, text excerpts, photographs) to substantiate analytic findings	5-13
**Discussion**			
Intergration with prior work, implications, transferability and contribution(s) to the field	#18	Short summary of main findings; explanation of how findings and conclusions connect to, support, elaborate on, or challenge conclusions of earlier scholarship; discussion of scope of application /	13-15

For peer review only

		generalizability; identification of unique contributions(s) to scholarship in a discipline or field	
Limitations	#19	Trustworthiness and limitations of findings	3,14
**Other**			
Conflicts of interest	#20	Potential sources of influence of perceived influence on study conduct and conclusions; how these were managed	15
Funding	#21	Sources of funding and other support; role of funders in data collection, interpretation and reporting	16

The SRQR checklist is distributed with permission of Wolters Kluwer © 2014 by the Association of American Medical Colleges. This checklist can be completed using https://www.goodreports.org/, a tool made by the EQUATOR Network in collaboration with Penelope.ai

For peer review only
